# Moving molecular medicine

**DOI:** 10.15252/emmm.201707746

**Published:** 2017-03-17

**Authors:** Philippe J Sansonetti

**Affiliations:** ^1^ Institut PasteurParisFrance

## Abstract

Incoming Chief Editor Philippe Sansonetti shares his vision of the role of the journal in the burgeoning areas of molecular medicine.

Following after clinical anatomy and experimental medicine, molecular medicine is medicine's third revolution in the last two centuries. Molecular medicine is a complex discipline that aims at deciphering the structural, molecular, and cellular pathways and signals underlying disease processes, identifying the possible genetic defects causing these dysfunctions, and thus to design molecular tools and interventions to diagnose, prevent and treat these diseases. Molecular medicine is in essence a multidisciplinary exercise that requires integrating physics, chemistry, biology, and increasingly bioinformatics and big data management.

EMBO could, of course, not ignore the molecular dimension of medicine. I attended the first EMBO sectorial meeting for molecular medicine organized by Frank Gannon in Dubrovnik in 2003. It was a revealing event, where cancer biologists, microbiologists and infection biologists, cardiovascular and kidney diseases experts, neuroscientists, gastroenterologists, hepatologists, endocrinologists, and experts in metabolic diseases realized that they shared a common language that transcended the various disciplines: the language of molecules, signals, and their regulation. At this meeting, I discovered the details of ubiquitination from talks on the genetic and molecular bases of the von Hippel‐Lindau syndrome and Fanconi anemia—since, covalent modification by small moieties like ubiquitin has become a dominant theme in infection signaling. This meeting laid the foundation for *EMBO Molecular Medicine*, even if it took a few years before we could celebrate its birth in 2009 at another memorable kickoff workshop, this time at EMBL.

This is also an opportunity to thank and congratulate Stefanie Dimmeler, my predecessor as Chief Editor, for firmly establishing the recognized position of *EMBO Molecular Medicine* in the field. She did a great job, together with two outstanding editors, Céline Carret and Roberto Buccione, to set the demanding standards that have secured the strong image of scientific quality and editing efficiency and transparency that characterize *EMBO Molecular Medicine*, and place it well in line with the other three EMBO Press journals.

As new Chief Editor, it will be my duty to pursue and buttress this successful editorial policy. I am strongly attached to this journal and its aim to promote molecular medicine, irrespective of its geographic source or subdiscipline, as long as contributions meet the EMBO standards of excellence.

Having said this, *EMBO Molecular Medicine* must continue to adapt to the fast‐changing landscape of molecular medicine. Geographically, the journal needs to have a more global outlook, and efforts will be made to strengthen the flux of American and Asian contributions. The journals' balance of disciplines also needs to be reequilibrated as some very active specialties remain under‐represented. This is the case, for example, with infection biology and metabolic diseases. Our senior editors will be mobilized to help restore visibility of these disciplines and we will collectively put the emphasis on commissioning high‐standard reviews with a true integrative dimension, particularly to set the stage for under‐represented disciplines.

We will also increase our efforts to make the journal more open and attractive to our community by encouraging submission of short “state‐of‐the‐art” commentaries and research papers by outstanding leaders, thereby permanently reevaluating the evolving boundaries of molecular medicine.

*EMBO Molecular Medicine* is our journal, I am honored and thrilled to take this Chief‐Editorial position, but also strongly committed to work with the editorial advisory board to make it an even more attractive and successful forum.

